# The assessment of self-stigmatization of patients with schizophrenia and complex approach to reduce it

**DOI:** 10.1192/j.eurpsy.2023.1055

**Published:** 2023-07-19

**Authors:** V. Mitikhin, T. A. Solokhina, A. I. Savushkina

**Affiliations:** 1 Department of Mental Health Support Systems Research Centre, Mental Health Research Centre; 2 Department of Labor and Social Protection of the Population, Moscow Service for Psychological Assistance to the Population, Moscow, Russian Federation

## Abstract

**Introduction:**

The negative consequences of the stigmatization of mental illness significantly impair health care system, society, patients and their families. It has been established, that more than 40% of patients with schizophrenia suffer from self-stigmatization (E. Brohan et al., 2010), what determines the relevance of research aimed at it’s reduction.

**Objectives:**

To assess the level, components of self-stigmatization and associated with it factors in patients with schizophrenia, receiving psychosocial treatment in the community; to propose and implement a complex of interventions for destigmatization.

**Methods:**

The battery of instruments was used: Self-stigmatization questionnaire (V.S. Yastrebov, I.I. Mikhailova et al., 2005), revealing the patient’s tendency to explain their problems in the main areas of psychosocial functioning as manifestations of the disease or the prejudice against them; Emotional intelligence questionnaire (D.V. Lyusin, 2006); Quality of life questionnaire (J.E. Ware et al., 1995); Montreal Cognitive Assessment (Z.S. Nasreddine, 1996). 40 patients with schizophrenia (ICD-10 F.20), receiving psychosocial treatment in a non-profit organization in community, were examined.

**Results:**

The overall level of self-stigmatization in the studied patients constituted 42.8% or an average level of self-stigmatization. Using Self-stigmatization questionnaire, nine components of self-stigmatization were revealed. The most pronounced indicators were in following components: “Reassessment of self-realization”, “Readiness to distance from the mentally ill in the social sphere”, “Reassessment of internal activity” (56.2%, 56.5%, 55.1% correspondingly). By the forms of self-stigmatization demonstrated that patients with autopsychic form (the justification of their failure by the disease) constituted the largest proportion or 41%. The compensatory form (denial of one’s incompetence with its exaggeration in other mentally ill people) and socio-reversive form (explaining incompetence by the prejudice against them) had similar rates in 29% and 30% of patients, correspondingly. Inverse strong correlations with some of scales of the Emotional intelligence questionnaire, Cognitive scale and the Quality of life questionnaire were established. Destigmatization training for patients with schizophrenia based on cognitive behavioral psychotherapy was worked out. A set of destigmatization interventions was proposed and implemented.

**Conclusions:**

A complex of different interventions taking into account the form of self-stigmatization and it’s main components, should be used. These interventions have to include psychoeducation, cognitive trainings, self-esteem trainings and special destigmatization trainings.

Keywords: schizophrenia, self-stigmatization, destigmatization trainings

**Disclosure of Interest:**

None Declared

